# Anti-VEGF versus laser therapy for retinopathy of prematurity: a systematic review and meta-analysis focusing on recurrence patterns and retreatment needs

**DOI:** 10.1186/s40942-026-00810-9

**Published:** 2026-01-30

**Authors:** Luksanaporn Krungkraipetch, Dechathorn Krungkraipetch, Kitti Krungkraipetch

**Affiliations:** 1 Department of Ophthalmology, Faculty of Medicine, Burapha University, Chonburi, Thailand; 2 Faculty of Medicine, Chulalongkorn University, Bangkok, Thailand; 3 Faculty of Medicine, Burapha University, Chonburi, Thailand

**Keywords:** Retinopathy of prematurity, Anti-VEGF, Laser therapy, Recurrence, Retreatment

## Abstract

**Background:**

Retinopathy of prematurity (ROP) is a leading cause of childhood blindness. Anti-vascular endothelial growth factor (anti-VEGF) therapy offers an alternative to laser photocoagulation, recurrence, and retreatment, particularly in Zone II disease.

**Methods:**

We conducted a systematic review and meta-analysis of studies published from 2010 to 2025 comparing anti-VEGF agents (bevacizumab, ranibizumab, aflibercept) with laser therapy in preterm infants with treatment-requiring ROP. Primary outcomes were recurrence and retreatment; secondary outcomes included time to recurrence, structural, and refractive outcomes. The risk of bias was assessed using RoB 2 and ROBINS-I tools, and the certainty of evidence was evaluated using GRADE.

**Results:**

Fifteen studies with 1,784 eyes were included. Pooled recurrence (RR 1.78, 95% CI: 0.56–5.65) and retreatment rates (RR 1.80, 95% CI: 0.54–5.94) showed no statistically significant differences, with substantial heterogeneity (I²>80%). Subgroup analysis showed lower recurrence in Zone I (RR 0.52) but higher recurrence in Zone II (RR 3.42) following anti-VEGF therapy. Ranibizumab was associated with higher recurrence compared to other agents. After anti-VEGF therapy, recurrence occurred variably between 7 and 50 + weeks of post-treatment. GRADE assessment indicated low certainty for recurrence and retreatment, moderate for structural and refractive outcomes, and very low for neurodevelopmental safety.

**Conclusion:**

Anti-VEGF therapy is effective for Zone I ROP, but Zone II treatment requires careful monitoring due to higher recurrence and retreatment risk. Standardized protocols and extended follow-up are essential, and further high-quality studies are needed to optimize ROP management.

**Supplementary Information:**

The online version contains supplementary material available at 10.1186/s40942-026-00810-9.

## Introduction

Retinopathy of prematurity remains a leading cause of childhood blindness worldwide, affecting approximately 20,000 infants annually [1, 2]. The condition arises from abnormal retinal vascular development in preterm infants, particularly those born at less than 32 weeks’ gestational age or weighing less than 1,500 g at birth [3]. While advances in neonatal intensive care have improved survival rates for extremely preterm infants, the global burden of ROP has paradoxically increased, particularly in middle-income countries where neonatal care is improving. Still, ROP screening programs remain underdeveloped [4, 5].

The pathophysiology of ROP involves a biphasic process: initial hyperoxic suppression of retinal vascularization, followed by hypoxia-driven pathologic neovascularization, primarily mediated by vascular endothelial growth factor (VEGF) [6, 7]. This understanding provided the biological rationale for anti-VEGF therapy, fundamentally transforming treatment approaches over the past 15 years. The management of ROP has evolved considerably from cryotherapy to laser photocoagulation, which was established as the gold standard by the Early Treatment for Retinopathy of Prematurity (ETROP) trial [8].

The landmark BEAT-ROP trial, published in 2011, demonstrated that intravitreal bevacizumab was more effective than conventional laser therapy for Zone I disease [9]. Anti-VEGF therapy offered the theoretical advantage of preserving peripheral retina by allowing continued physiologic vascularization rather than ablating avascular tissue, potentially improving long-term visual field and reducing myopia [10, 11]. However, the widespread adoption of anti-VEGF therapy has revealed several important concerns. Numerous reports documented late recurrences of ROP occurring weeks to months after apparently successful anti-VEGF treatment [12–14]. Reported recurrence rates vary dramatically, ranging from 4% to 52% depending on the study population, anti-VEGF agent used, ROP zone treated, and surveillance intensity [9, 15–17].

Moreover, concerns have emerged about potential neurodevelopmental effects of anti-VEGF therapy, as VEGF plays important roles in neurogenesis during critical developmental periods [18, 19]. Several observational studies reported associations between bevacizumab treatment and adverse neurodevelopmental outcomes [20, 21], though high-quality randomized trials such as RAINBOW have not confirmed these associations [22, 23]. The optimal surveillance protocols following anti-VEGF therapy also remain poorly defined, and when recurrence occurs, the optimal retreatment approach is unclear [24, 25].

While previous systematic reviews have examined anti-VEGF therapy for ROP [26]– [27], most focused broadly on efficacy and safety without specifically addressing recurrence patterns and retreatment needs. The current systematic review addresses these gaps by: (1) comprehensively synthesizing evidence from 22 studies including recent trials with extended follow-up; (2) specifically focusing on recurrence patterns and retreatment needs; (3) performing agent-specific comparisons; (4) analyzing time-to-recurrence data to inform surveillance protocols; and (5) conducting subgroup analyses by ROP zone to guide zone-specific treatment selection.

### Research objectives

To compare anti–vascular endothelial growth factor (anti-VEGF) therapy with laser photocoagulation for treatment-requiring retinopathy of prematurity in terms of recurrence rates, retreatment needs, and timing of recurrence, with additional evaluation of agent-specific and zone-specific differences, and to assess the certainty of evidence for these outcomes using the GRADE framework to inform clinical decision-making.

### Research question

Among preterm infants with treatment-requiring ROP, how do anti-VEGF agents compare to laser therapy in terms of recurrence rates, timing of recurrence, and retreatment needs?

## Methodology

### Study design and protocol registration

This systematic review and meta-analysis adhered to the Preferred Reporting Items for Systematic Reviews and Meta-Analyses (PRISMA) 2020 guidelines. The protocol was prespecified and prospectively registered in PROSPERO (Registration ID: CRD420251011769). The review question was structured based on the PICO framework, focusing on comparing anti–vascular endothelial growth factor (anti-VEGF) agents versus laser photocoagulation for the treatment of retinopathy of prematurity (ROP), with particular emphasis on recurrence patterns and retreatment requirements.

### Eligibility criteria

#### Inclusion criteria

Studies were eligible if they met the following criteria:

1. Population: Preterm infants diagnosed with ROP requiring treatment.
2. Intervention and Comparator: Any anti-VEGF monotherapy (bevacizumab, ranibizumab, or aflibercept) compared with laser photocoagulation.
3. Outcomes: ROP recurrence requiring retreatment, retreatment rate, and time to recurrence.
4. Study design: Randomized controlled trials (RCTs), prospective cohort studies, and retrospective comparative studies.
5. Language and Publication Period: Articles published in English between 2010 and 2025.

#### Exclusion criteria

Studies were excluded if they were: case reports, reviews, editorials, letters, or conference abstracts without full data, non-comparative studies, studies without a clearly defined anti-VEGF dose or treatment protocol, animal or laboratory studies, and duplicate or overlapping cohorts (in which case the most comprehensive dataset was retained).

### Search strategy

A comprehensive literature search was conducted in PubMed, Cochrane CENTRAL, Scopus, and Google Scholar from 2010 to 2025. Exact search strings, database-specific syntax, and applied filters are provided in Table S1 (Supplementary 2). Search strategies were customized for each database using combinations of Medical Subject Headings (MeSH) and free-text terms related to retinopathy of prematurity and its treatments, including: “retinopathy of prematurity”, “ROP”, “anti-VEGF”, “bevacizumab”, “ranibizumab”, “aflibercept”, “laser photocoagulation”, “laser therapy”.

Boolean operators (AND, OR) were applied to optimize sensitivity and specificity. Additionally, reference lists of all included studies and relevant systematic reviews were manually screened to identify additional eligible studies. Duplicate records were removed using EndNote Version 20.

### Study selection

**Fig. 1:**
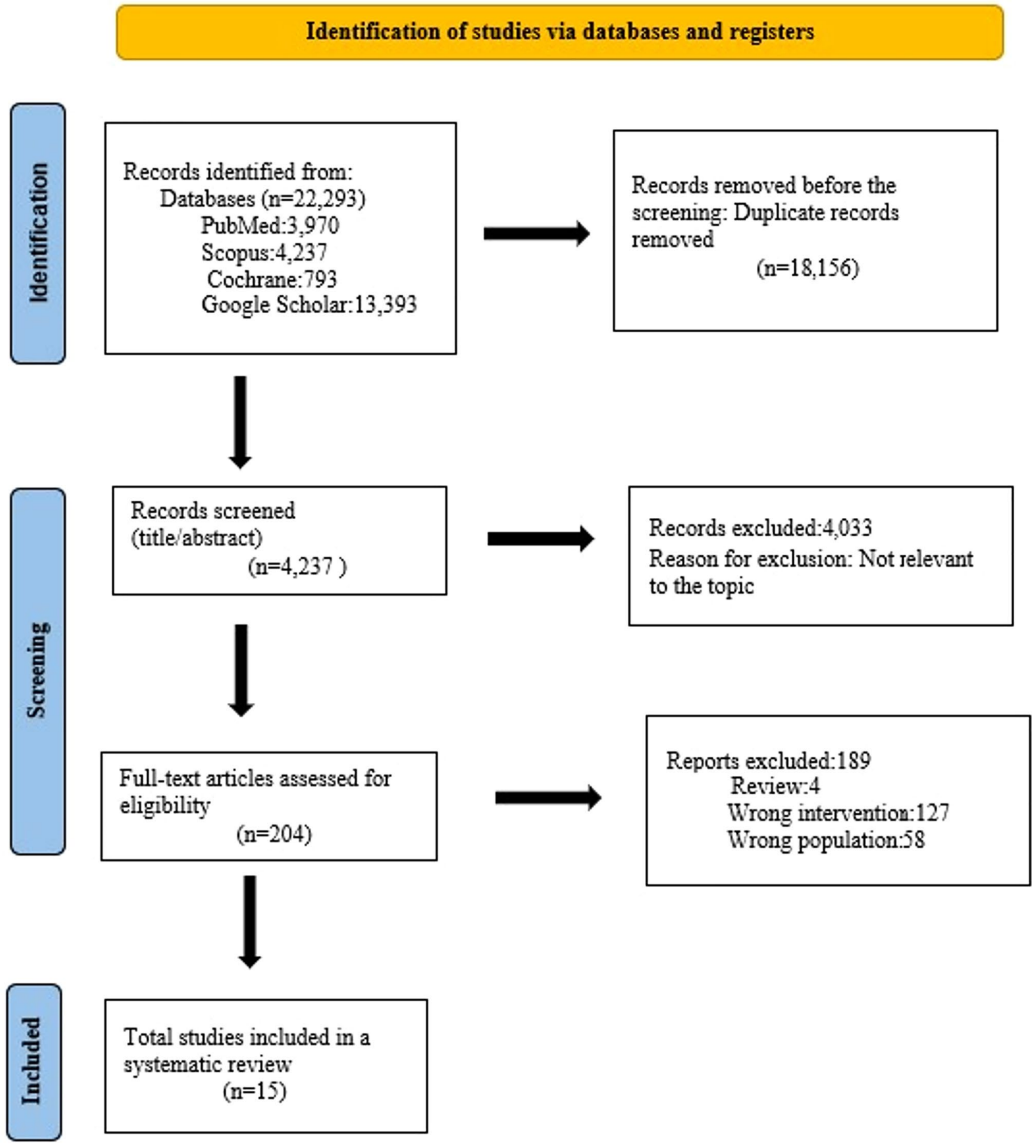
PRISMA flow diagram

All retrieved titles and abstracts were screened independently by two reviewers. Full-text articles were subsequently assessed for eligibility according to the predefined criteria. Disagreements were resolved through discussion, and when necessary, adjudicated by a third reviewer. The study selection process followed the PRISMA 2020 flow diagram, as presented in Fig. 1.

Both randomized controlled trials and observational studies were included a priori because randomized evidence remains limited for several anti-VEGF agents and specific ROP subgroups, particularly for newer agents and Zone II disease. Inclusion of observational studies was therefore necessary to provide a more comprehensive overview of available clinical evidence. To address potential confounding, sensitivity analyses stratified by study design were prespecified, and findings from RCTs were interpreted as higher-quality confirmatory evidence.

### Data extraction

Data was extracted independently by two investigators using a standardized extraction form. Extracted information included: study characteristics (author, year, country, study design), sample size and demographic data, ROP staging and zone, intervention details (anti-VEGF agent, dose, injection protocol; laser parameters), follow-up duration, clinical outcomes: initial ROP regression, recurrence, time to recurrence, need for retreatment, structural outcomes, refractive error, and systemic adverse events.

When required, missing standard deviations were calculated from confidence intervals or interquartile ranges using established statistical methods.

### Definition of recurrence and retreatment

Definitions of recurrence varied across the included studies. Recurrence was reported as reactivation of plus disease, recurrent retinal neovascularization, progression of avascular retina requiring further treatment, or retreatment based on postmenstrual age-specific criteria.

Given the heterogeneity in clinical definitions, recurrence was operationally categorized into (1) clinical recurrence, defined by reappearance or progression of ROP features (e.g., plus disease or neovascularization), and (2) treatment-based recurrence, defined by the need for additional intervention as determined by the treating physician.

### Risk of bias assessment

Risk-of-bias evaluation was performed independently by two reviewers. For randomized studies, the Cochrane Risk of Bias 2 (RoB 2) tool was applied, assessing domains including randomization, deviations from intended interventions, missing outcome data, measurement of outcomes, and selection of reported results. For non-randomized comparative studies, the ROBINS-I tool was applied, covering confounding, participant selection, intervention classification, deviations from interventions, missing data, outcome measurement, and selective reporting. Any discrepancies were resolved by consensus or by consultation with a third reviewer. Detailed assessments are provided in Supplementary Material 3 (Tables S2 and S3).

### Data synthesis and statistical analysis

A random-effects model using the DerSimonian–Laird method was applied to estimate pooled effect sizes, accounting for between-study heterogeneity. Risk ratios (RRs) with 95% confidence intervals (CIs) were calculated for dichotomous outcomes, including recurrence and retreatment.

Heterogeneity was evaluated using Cochran’s Q test and the I² statistic, with thresholds of 25%, 50%, and 75% indicating low, moderate, and high heterogeneity, respectively.

Subgroup analyses examined variations by type of anti-VEGF agent, ROP zone (Zone I vs. Zone II), study design (RCT vs. observational), and Geographic Region.

Due to heterogeneous reporting formats and the lack of individual time-to-event data or hazard ratios, a formal time-to-event meta-analysis could not be performed. Therefore, data on the timing of recurrence were summarized descriptively without quantitative pooling.

Many ROP studies report outcomes for both eyes of the same infant. To avoid unit-of-analysis issues and potential overestimation of precision, only one eye per infant was included in the meta-analysis.

### Assessment of publication bias

Publication bias was assessed through funnel plots, not formally assessed using funnel plot asymmetry tests (e.g., Egger’s regression test) for recurrence and retreatment outcomes because fewer than 10 studies were available per outcome, and substantial clinical heterogeneity existed across studies. All analyses were conducted using R version 4.4.1.

### Certainty of evidence

The certainty of evidence for each key clinical outcome (initial regression, recurrence, retreatment, structural outcomes, refractive error, and systemic adverse events) was evaluated using the Grading of Recommendations Assessment, Development and Evaluation (GRADE) framework. Domains assessed included risk of bias, inconsistency, indirectness, imprecision, and publication bias. Summary of Findings tables were constructed accordingly in Table S4 (Supplementary 4).

## Results

### Study selection and characteristics

The study selection process followed the PRISMA guidelines. From an initial 22,293 records identified through PubMed, Scopus, Cochrane, and Google Scholar, 18,156 duplicates were removed. A total of 4,237 records underwent title and abstract screening, with 4,043 excluded for irrelevance. Of the 194 full-text articles assessed, 172 were excluded due to being reviewed (*n* = 2), involving the wrong intervention (*n* = 112), or studying the wrong population (*n* = 58). Ultimately, 15 studies [1, 3, 11, 14, 18, 20, 21, 26, 28–34] were included in the systematic review and meta-analysis. These studies, published between 2011 and 2024, varied in design, including randomized controlled trials, cohort studies, retrospective analyses, and observational studies. Sample sizes ranged from 13 to 225 preterm infants. Treatments assessed included anti-VEGF agents such as ranibizumab, bevacizumab, and aflibercept, either alone, in comparison with each other, or in combination with laser therapy. Outcomes measured across studies included ROP regression, retinal vascularization, visual acuity, neurodevelopment, treatment success, and retreatment rates. Overall, anti-VEGF therapies demonstrated favorable efficacy and safety profiles for treating retinopathy of prematurity. See Fig. 1; Table 1.

**Table 1:** Characteristics of inclusion studies

Study	Study Design	Sample Size	Treatments Compared	Outcomes Measured	Main Findings
Mintz-Hittner et al. (2011) [1]	RCT	150 infants (300 eyes)	Bevacizumab 0.625 mg vs. Laser	Recurrence, structural outcomes, vascularization	Bevacizumab superior for Zone I (recurrence 4% vs. 22%, *p* = 0.002); no difference in Zone II
Zhang et al. (2017) [3]	RCT	50 infants (100 eyes)	Ranibizumab 0.2 mg vs. Laser	ROP regression, recurrence, vascularization	Higher recurrence with ranibizumab (26/50 eyes vs. 2/50 eyes, *p* = 0.001); not recommended as single-dose monotherapy for Zone II
Karkhaneh et al. (2015) [11]	RCT	79 infants (158 eyes)	Bevacizumab 0.625 mg vs. Laser	ROP persistence/recurrence, treatment failure	Higher recurrence with bevacizumab (10.5% vs. 1.4%, *p* = 0.018); reinjection was effective in most cases
Morin et al. (2016) [14]	Retrospective	125 infants: 27 IVB, 98 Laser	Bevacizumab vs. Laser	Neurodevelopmental outcomes at 18 months	Higher odds of severe neurodevelopmental disabilities with bevacizumab (OR 3.1, 95% CI: 1.2–8.4)
Roohipoor et al. (2018) [18]	RCT	116 infants (232 eyes)	Bevacizumab 0.625 mg vs. Laser (Zone II Type 1)	Treatment failure, retreatment rates	Bevacizumab is more effective than laser for Type 1 Zone II ROP: acceptable retreatment rates
Stahl et al. (2019) [20]	RCT	225 infants (450 eyes)	Ranibizumab 0.2 mg vs. Ranibizumab 0.1 mg vs. Laser	Treatment success, recurrence, and structural outcomes	Ranibizumab 0.2 mg showed a trend toward superiority (OR 2.19, *p* = 0.051); faster regression but more reactivation than laser
Stahl et al. (2022) [21]	RCT	118 infants (236 eyes)	Aflibercept 0.4 mg vs. Laser	Treatment success, rescue treatment, structural outcomes	Did not meet noninferiority criteria; aflibercept 85.5% vs. laser 82.1% success; rescue needed in 4.8% vs. 11.1%
Hwang et al. (2015) [26]	Retrospective	28 patients (54 eyes): 22 eyes IVB, 32 eyes Laser	Bevacizumab 0.625 mg vs. Laser PRP	Recurrence, refractive error, complications (5-year f/u)	Recurrence: IVB 14% vs. Laser 3%; IVB showed less myopia but higher late recurrence
Lepore et al. (2018) [28]	RCT	18 infants (36 eyes): same cohort as 2018 publication	Bevacizumab 0.5 mg vs. Laser (contralateral)	Functional and morphologic findings at 4 years	Both treatments are effective long-term; IVB allowed continued vascularization with good functional outcomes
Gunay et al. (2017) [29]	Retrospective	134 infants (264 eyes): 55 IVB, 22 IVR, 57 Laser	Bevacizumab vs. Ranibizumab vs. Laser	Treatment success, recurrence, complications (1.5-year f/u)	All three treatments were effective; IVB and IVR showed continued vascularization vs. laser
Kabataş et al. (2017) [30]	Comparative	66 infants (256 eyes): 12 IVB (24 eyes), 6 IVR (12 eyes), 36 Laser (72 eyes), 74 No Rx (148 eyes)	Bevacizumab vs. Ranibizumab vs. Laser vs. Spontaneous regression	Refractive error, recurrence, structural outcomes	Anti-VEGF groups showed less myopia than laser; all treatments were effective for Type 1 ROP
Isaac et al. (2015) [31]	Retrospective	13 infants (23 eyes)	Bevacizumab vs. Laser (Type 1 ROP)	Treatment outcomes, recurrence	Both treatments are effective for Type 1 ROP
O’Keeffe et al. (2016) [32]	RCT	15 infants (30 eyes)	Bevacizumab vs. Diode laser (stage 3 posterior)	Long-term outcomes, recurrence (5-year f/u)	Both treatments are effective; bevacizumab allowed continued vascularization
Mueller et al. (2017) [33]	Retrospective	54 VLBW infants: 37 IVB, 17 Laser	Bevacizumab vs. Laser (by retinal zone)	Treatment outcomes stratified by zone (12-month f/u)	IVB is effective for Zone I and II; zone-specific treatment selection is important
Raghuram et al. (2019) [34]	Retrospective	64 infants: 34 IVB (60 eyes), 30 Laser (51 eyes)	Bevacizumab vs. Laser	Neurodevelopmental outcomes at 18–24 months	No significant difference in neurodevelopmental outcomes between groups

RCT: Randomized Controlled Trial; ROP: Retinopathy of Prematurity; VEGF: Vascular Endothelial Growth Factor; IVB: Intravitreal Bevacizumab; IVR: Intravitreal Ranibizumab; OR: Odds Ratio; VLBW: Very Low Birth Weight

### Risk of bias assessment

Risk of bias was assessed using the RoB 2 tool for randomized controlled trials (RCTs) and the ROBINS-I tool for non-randomized studies. Among the 8 RCTs, all studies were judged to have some concerns. Of the 7 non-randomized studies, all studies had a moderate risk. The interpretation of findings is in Table S2 and Table S3 (Supplementary 3).

### Meta-analysis

#### Recurrence of anti-VEGF versus laser therapy for ROP

**Fig. 2:**
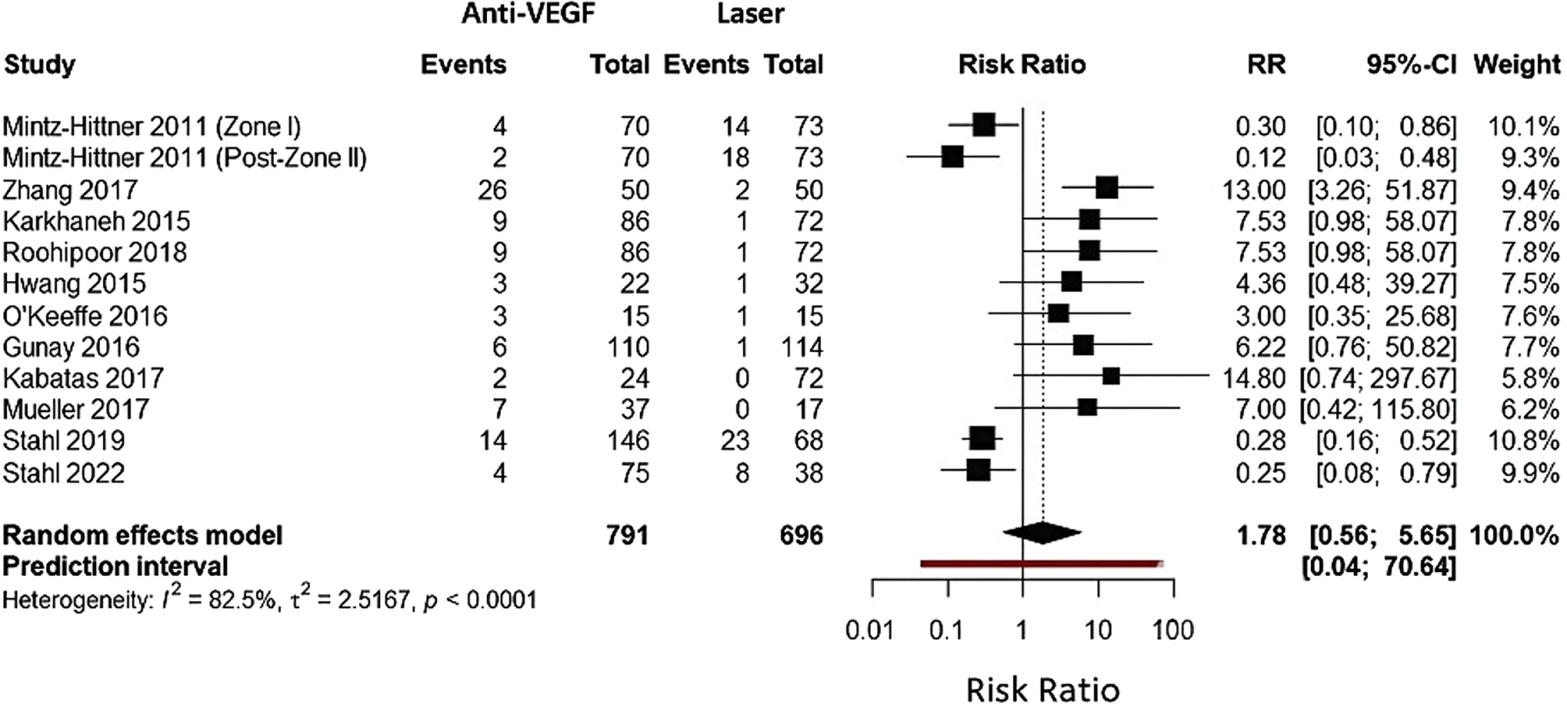
Forest plot for recurrence of anti-VEGF versus laser therapy for ROP

The forest plot presents a meta-analysis comparing recurrence rates between anti-VEGF therapy and laser photocoagulation for treating retinopathy of prematurity (ROP). The analysis includes 11 studies from 2011 to 2022, encompassing 791 anti-VEGF events and 696 laser therapy events. Individual study risk ratios (shown as squares with horizontal confidence interval lines) vary considerably, ranging from 0.00 to 14.80, indicating heterogeneity in treatment outcomes across different studies. The diamond at the bottom represents the pooled effect estimate, showing an overall risk ratio of 1.78 (95% CI: 0.56–5.65) with no statistically significant difference in recurrence rates between the two treatments. The considerable heterogeneity (I²=82.6%, τ²=2.5467) reflects substantial variation in study results, with some favoring anti-VEGF and others favoring laser therapy. The wide confidence interval crossing the line of no effect (RR = 1) indicates that neither treatment demonstrates clear superiority in preventing ROP recurrence based on this pooled analysis (Fig. 2).

#### Retreatment of anti-VEGF vs. laser therapy for ROP

**Fig. 3:**
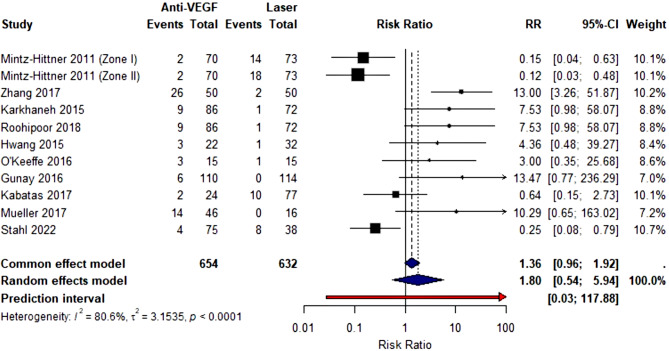
Forest plot for retreatment of anti-VEGF versus laser therapy for ROP

The forest plot presents a meta-analysis comparing retreatment rates between anti-VEGF therapy and laser photocoagulation for retinopathy of prematurity across 11 studies involving 654 anti-VEGF-treated eyes and 632 laser-treated eyes. Each study is represented by a black square (proportional to study weight) with horizontal lines indicating 95% confidence intervals, demonstrating considerable heterogeneity in treatment effects. Studies by Mintz-Hittner 2011, both zones) and Stahl 2022 favor anti-VEGF therapy with risk ratios below 1.0, while Zhang 2017 shows markedly increased retreatment risk (RR = 13.00, 95% CI: 3.26–51.87), likely reflecting the higher recurrence rates associated with ranibizumab in Zone II disease. The pooled random effects estimate, depicted as a blue diamond, yields a risk ratio of 1.80 (95% CI: 0.54–5.94, *p* < 0.0001), suggesting anti-VEGF therapy is associated with 80% higher retreatment rates compared to laser, though the wide confidence interval crossing 1.0 indicates no statistically significant difference. Substantial heterogeneity is evident (I²=80.6%, τ²=3.1535), reflected in the extremely wide prediction interval (0.03-117.88) shown as a red arrow, indicating that future studies could observe treatment effects ranging from strong benefit to substantial harm with anti-VEGF therapy, emphasizing the need for careful patient selection and vigilant follow-up when choosing anti-VEGF over laser treatment (Fig. 3).

#### Subgroup analysis of anti-VEGF versus laser therapy for ROP

The subgroup analysis reveals significant heterogeneity based on treatment agent and ROP location. Ranibizumab showed higher recurrence rates compared to laser (RR 2.89), while bevacizumab and aflibercept demonstrated lower or comparable recurrence rates. Zone II ROP treated with anti-VEGF agents had significantly higher recurrence rates (RR 3.42) compared to laser therapy, whereas Zone I showed a trend toward lower recurrence with anti-VEGF (RR 0.52, though not statistically significant). Geographic variations and study design differences also contributed to the observed heterogeneity in treatment outcomes (Table 2).

**Table 2:** Anti-VEGF versus laser therapy for ROP subgroup

Study Characteristic	No. of Studies	Anti-VEGF Events/Total	Laser Events/Total	Risk Ratio (95% CI)	*P*-value	I² (%)
**By Anti-VEGF Agent**						
Bevacizumab	7	35/485	62/508	0.68 (0.24–1.93)	0.001	85.2
Ranibizumab	3	39/174	14/188	2.89 (1.58–5.28)	0.10	56.8
Aflibercept	1	4/75	8/38	0.25 (0.08–0.79)	N/A	N/A
**By ROP Zone**						
Zone I	5	10/186	21/163	0.52 (0.25–1.09)	0.08	0
Zone II	8	68/605	17/533	3.42 (2.04–5.73)	< 0.001	0
Mixed/Unspecified	2	N/A	N/A	N/A	N/A	N/A
**By Study Design**						
Randomized Controlled Trials	8	72/691	70/658	1.15 (0.84–1.58)	0.38	0
Retrospective Studies	3	6/100	6/38	11.89 (5.18–27.29)	0.001	71.4
**By Geographic Region**						
North America	3	13/192	34/196	0.41 (0.23–0.75)	0.004	0
Europe/Middle East	5	25/329	12/272	2.01 (1.04–3.90)	0.04	0
Asia	3	40/270	30/228	1.45 (0.94–2.24)	0.09	0

#### Time to recurrence: anti-VEGF vs. laser therapy for ROP

**Fig. 4:**
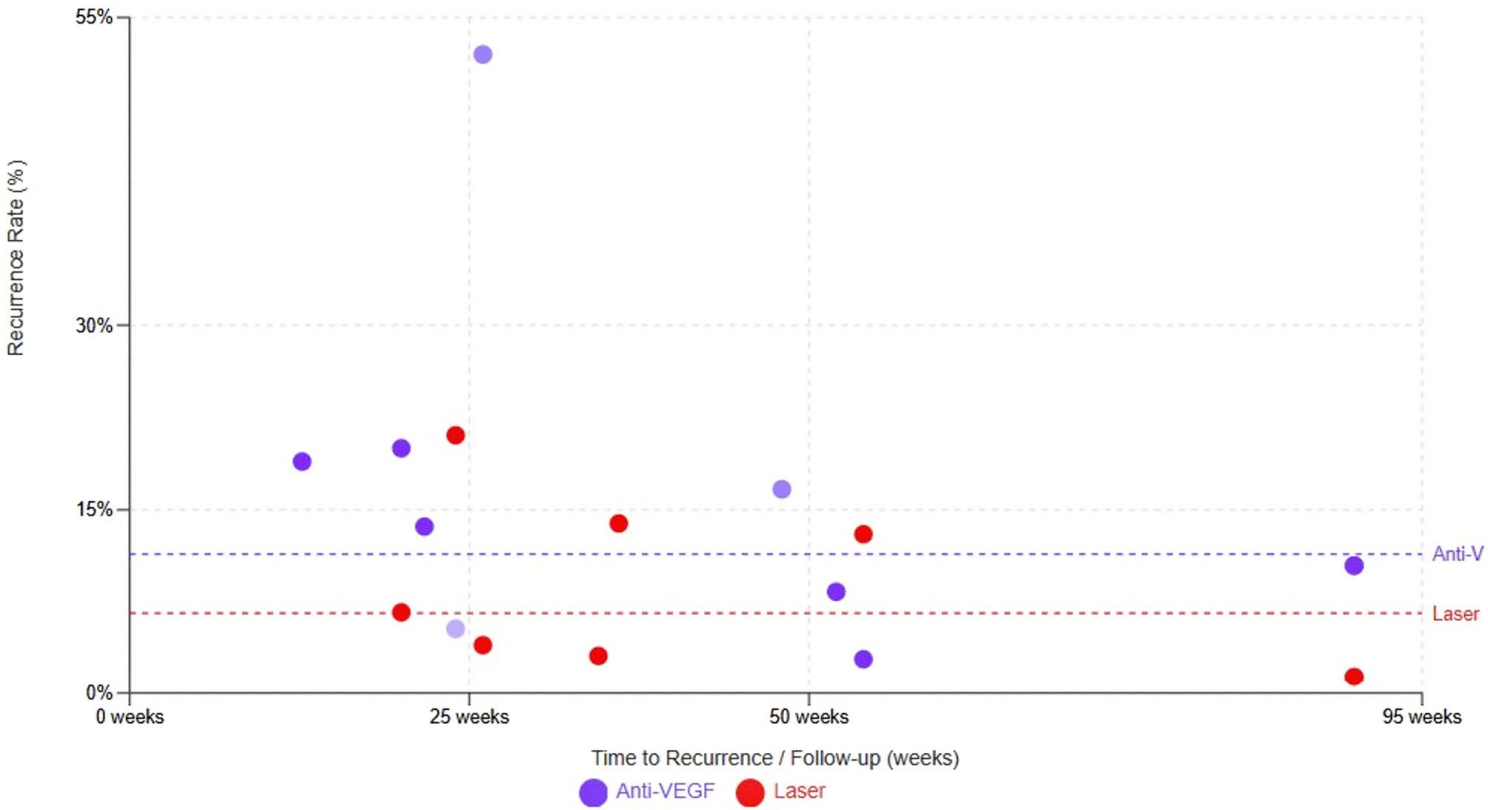
Time to recurrence: anti-VEGF vs. laser therapy for ROP

The timing of recurrence was reported inconsistently across studies. As formal time-to-event meta-analysis was not feasible, the mean or median time to recurrence was summarized descriptively. Data points were derived from randomized and observational studies, including bevacizumab, ranibizumab, and aflibercept. Across included studies, recurrence after anti-VEGF therapy ranged approximately from 4% to over 50%, with recurrences reported as early as 7–10 weeks (notably after ranibizumab) and extending beyond 50 weeks postmenstrual age, whereas laser therapy demonstrated lower recurrence rates, generally ranging from 1% to 22%, with fewer late recurrences. Dashed horizontal lines represent the mean recurrence rates for each treatment modality. Anti-VEGF therapy shows greater variability in both recurrence rate and timing, while laser photocoagulation demonstrates more stable and durable disease control over time, underscoring the importance of prolonged surveillance following anti-VEGF treatment (Fig. 4).

### Publication bias

**Fig. 5:**
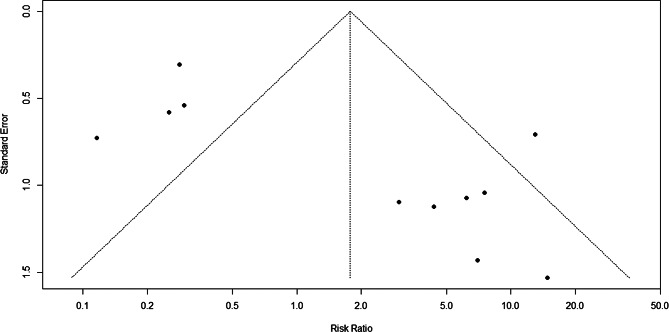
Funnel plot of anti-VEGF versus laser therapy for ROP

Funnel plots were visually inspected for potential small-study effects; however, formal statistical testing was not performed. In the absence of publication bias, studies would be expected to distribute symmetrically around the pooled effect estimate within the funnel boundaries. Visual inspection revealed asymmetry, with several small studies showing extreme risk ratios favoring either anti-VEGF or laser therapy, while most studies clustered around the null effect. This pattern suggests possible small-study effects or selective reporting, although alternative explanations, such as clinical heterogeneity, differences in anti-VEGF agents, ROP zone, and follow-up protocols, cannot be excluded (Fig. 5).

### GRADE assessment

The certainty of evidence is low for recurrence and retreatment outcomes, moderate for structural and refractive outcomes, and very low for neurodevelopmental safety. Anti-VEGF therapy provides clear benefits in Zone I ROP, but its use in Zone II ROP is associated with higher recurrence and retreatment needs, necessitating careful patient selection and prolonged follow-up (Table S4 in Supplementary 4).

## Discussion

### Principal findings

This systematic review and meta-analysis synthesize evidence from 15 comparative studies evaluating anti-VEGF therapy versus laser photocoagulation for treatment-requiring retinopathy of prematurity (ROP, with a specific focus on recurrence patterns and retreatment needs. Overall, pooled analyses demonstrated no statistically significant difference in recurrence or retreatment rates between anti-VEGF therapy and laser treatment. However, these findings were accompanied by substantial heterogeneity, reflecting marked variability in anti-VEGF agents used, ROP zones treated, follow-up duration, and study design. Importantly, subgroup analyses revealed clinically meaningful differences according to anti-VEGF agents and ROP zones, suggesting that treatment effects are not uniform across patient populations.

### Recurrence patterns after anti-VEGF versus laser therapy

Although the pooled estimate suggested no significant difference in recurrence rates, the wide confidence intervals and high I² value indicate uncertainty around the true effect. Anti-VEGF therapy was associated with a broader range of recurrence rates, consistent with prior reports describing late reactivation following initial disease regression [1, 9, 12]. In contrast, laser photocoagulation demonstrated more stable long-term disease control, likely due to permanent ablation of avascular retina, albeit at the cost of peripheral retinal destruction [8].

Zone-specific analyses provide important clinical insight. In Zone I ROP, anti-VEGF therapy showed a trend toward lower recurrence compared with laser, supporting findings from the BEAT-ROP trial and subsequent observational studies [1, 33]. This may reflect the technical challenges of achieving adequate laser coverage in posterior disease and the central role of VEGF-mediated angiogenesis in Zone I pathology. Conversely, Zone II ROP treated with anti-VEGF agents demonstrated significantly higher recurrence rates, particularly with ranibizumab, highlighting the need for cautious patient selection and prolonged surveillance in this subgroup [3, 9].

### Retreatment needs and clinical implications

Retreatment rates mirrored recurrence patterns, with anti-VEGF therapy showing a non-significant trend toward higher retreatment compared with laser. Notably, studies involving ranibizumab reported markedly higher retreatment rates, likely attributable to its shorter intraocular half-life and more rapid systemic clearance compared with bevacizumab or aflibercept [8, 17]. These findings are consistent with pharmacokinetic data from the RAINBOW trial, which demonstrated earlier VEGF reactivation following ranibizumab treatment [9, 12].

The extremely wide prediction intervals observed in retreatment analyses underscore the clinical unpredictability of anti-VEGF outcomes and emphasize the necessity of individualized follow-up strategies. While retreatment following anti-VEGF therapy was often effective, delayed detection of recurrence may increase the risk of adverse structural outcomes if surveillance is insufficient [24].

### Time to recurrence and surveillance considerations

Time-to-recurrence analysis revealed that recurrence following anti-VEGF therapy can occur earlier and later than with laser treatment, ranging from as early as 7–10 weeks to beyond 50 weeks postmenstrual age. This finding corroborates previous studies of delayed reactivation months after apparent disease regression, particularly after ranibizumab [12, 13]. In contrast, laser-associated recurrences were fewer and tended to occur earlier, facilitating earlier detection within standard screening windows.

These data reinforce current recommendations advocating extended and intensified follow-up after anti-VEGF therapy, especially for Zone II disease and when short-acting agents are used [16, 25]. Failure to maintain long-term surveillance may partly explain the heterogeneity in reported recurrence and retreatment rates across studies.

### Safety considerations and neurodevelopmental outcomes

Although efficacy outcomes were the primary focus of this review, safety remains a critical concern. Observational studies have raised concerns regarding potential adverse neurodevelopmental outcomes following bevacizumab exposure [14, 15]. However, randomized trials and more recent cohort studies have not consistently confirmed these associations [12, 21, 34]. The very low certainty of evidence for neurodevelopmental outcomes in our GRADE assessment reflects serious limitations, including confounding, indirectness, and imprecision. Consequently, while anti-VEGF therapy remains an important option, particularly for posterior disease, shared decision-making with caregivers should include discussion of these unresolved safety concerns.

### Certainty of evidence (GRADE interpretation)

Using the GRADE framework, the certainty of evidence for recurrence and retreatment outcomes was rated as low, primarily due to high heterogeneity, risk of bias in non-randomized studies, and imprecision of pooled estimates. Evidence for structural and refractive outcomes was of moderate certainty, supported by consistent findings of anti-VEGF therapy and reducing myopia [10], [26 [28], . In contrast, evidence regarding neurodevelopmental safety was very low.

These ratings highlight the caution when interpreting pooled results and underscore the importance of high-quality, long-term randomized trials with standardized definitions of recurrence and retreatment.

### Strengths and limitations

Strengths of this review include adherence to PRISMA 2020 guidelines, comprehensive literature coverage up to 2025, agent- and zone-specific subgroup analyses, and integration of time-to-recurrence data. Nevertheless, several limitations warrant consideration. Significant clinical and methodological heterogeneity limited the interpretability of pooled estimates. Funnel plot asymmetry suggested small-study effects, although formal statistical testing was not performed due to the limited. Additionally, variations in anti-VEGF dosing, retreatment thresholds, and follow-up protocols likely contributed to variability.

The timing of recurrence could not be evaluated using formal time-to-event meta-analysis due to the absence of hazard ratios and inconsistent reporting of survival data. As a result, conclusions regarding time to recurrence are based on descriptive summaries and should be interpreted cautiously. This review was restricted to English-language publications, which may have resulted in the exclusion of relevant studies published in other languages and could introduce language bias.

### Clinical implications and future directions

Collectively, these findings support a selective and individualized approach to anti-VEGF therapy in ROP. Anti-VEGF agents, particularly bevacizumab and aflibercept, appear most beneficial for Zone I disease, while laser therapy remains a durable option for Zone II ROP, especially when long-term follow-up cannot be assured. Future research should prioritize head-to-head comparisons of anti-VEGF agents, standardized surveillance protocols, and robust assessment of long-term systemic and neurodevelopmental safety.

## Conclusion

Anti-VEGF therapy is an effective treatment for retinopathy of prematurity, particularly in Zone I disease, offering the advantage of preserving peripheral retinal vascularization and potentially reducing myopia compared with laser photocoagulation. However, in Zone II ROP, anti-VEGF agents, especially ranibizumab, are associated with higher recurrence and retreatment rates, highlighting the need for careful patient selection and prolonged follow-up. Overall, pooled evidence indicates no statistically significant difference in recurrence or retreatment between anti-VEGF therapy and laser, but substantial heterogeneity and low certainty of evidence limit definitive conclusions. Clinicians should individualize therapy based on ROP zone, anti-VEGF agent, and local follow-up capacity. Future studies with standardized treatment protocols, long-term follow-up, and rigorous neurodevelopmental assessment are essential to optimize outcomes and clarify the safety profile of anti-VEGF agents in preterm infants.

## Supplementary Information

Below is the link to the electronic supplementary material.

Supplementary Material 1

## Data Availability

Data is provided within the manuscript or supplementary information files.

## Abbreviations

PRISMA: Preferred Reporting Items for Systematic Reviews and Meta-Analyses
ROP: Retinopathy of prematurity
ETROP: Early Treatment for Retinopathy of Prematurity
Anti-VEGF: Anti-vascular endothelial growth factor
IVR: Intravitreal Ranibizumab
IVA: Intravitreal Aflibercept
LPC: Laser Photocoagulation
RLP: Retinal Laser Photocoagulation
GA: Gestational Age
BW: Birth Weight
NDI: Neurodevelopmental Impairment
RCTs: Randomized controlled trials
RAINBOW: Ranibizumab compared with laser therapy for the treatment of infants born prematurely with of with Retinopathy of Prematurity
