# Supplementary material for: Anti-VEGF versus laser therapy for retinopathy of prematurity: a systematic review and meta-analysis focusing on recurrence patterns and retreatment needs
Source: Int J Retina Vitreous. 2026 Jan 30;12:30. doi: 10.1186/s40942-026-00810-9 (PMC12875000; doi:10.1186/s40942-026-00810-9)
Supplement: Supplementary file 1 — Supplementary Material 1 [file 40942_2026_810_MOESM1_ESM.docx]

**Supplementary Material**

**1. Supplementary Material 1: PRISMA 2020 checklist**

**2. Supplementary Material 2: Full Search Strategy**

**3.** **Supplementary Material 3: Risk of Bias**

**4. Supplementary Material 4: Certainty of Evidence (GRADE)**

**Supplementary Material 1: PRISMA 2020 checklist**

| **Section and Topic** | **Item #** | **Checklist item** | **Location where item is reported** |
| --- | --- | --- | --- |
| **TITLE** | | |  |
| Title | 1 | Identify the report as a systematic review. | Page 1 |
| **ABSTRACT** | | |  |
| Abstract | 2 | See the PRISMA 2020 for Abstracts checklist. | Page 2 |
| **INTRODUCTION** | | |  |
| Rationale | 3 | Describe the rationale for the review in the context of existing knowledge. | Page 3 |
| Objectives | 4 | Provide an explicit statement of the objective(s) or question(s) the review addresses. | Page 4 |
| **METHODS** | | |  |
| Eligibility criteria | 5 | Specify the inclusion and exclusion criteria for the review and how studies were grouped for the syntheses. | Page 5 |
| Information sources | 6 | Specify all databases, registers, websites, organisations, reference lists and other sources searched or consulted to identify studies. Specify the date when each source was last searched or consulted. | Page 6 |
| Search strategy | 7 | Present the full search strategies for all databases, registers and websites, including any filters and limits used. | Page 6 and Supplementary 2 |
| Selection process | 8 | Specify the methods used to decide whether a study met the inclusion criteria of the review, including how many reviewers screened each record and each report retrieved, whether they worked independently, and if applicable, details of automation tools used in the process. | Page 6 |
| Data collection process | 9 | Specify the methods used to collect data from reports, including how many reviewers collected data from each report, whether they worked independently, any processes for obtaining or confirming data from study investigators, and if applicable, details of automation tools used in the process. | Page 6 |
| Data items | 10a | List and define all outcomes for which data were sought. Specify whether all results that were compatible with each outcome domain in each study were sought (e.g. for all measures, time points, analyses), and if not, the methods used to decide which results to collect. | Page 6 |
|  | 10b | List and define all other variables for which data were sought (e.g. participant and intervention characteristics, funding sources). Describe any assumptions made about any missing or unclear information. | Page 6 |
| Study risk of bias assessment | 11 | Specify the methods used to assess risk of bias in the included studies, including details of the tool(s) used, how many reviewers assessed each study and whether they worked independently, and if applicable, details of automation tools used in the process. | Page 6- 7 |
| Effect measures | 12 | Specify for each outcome the effect measure(s) (e.g. risk ratio, mean difference) used in the synthesis or presentation of results. | Page 7 |
| Synthesis methods | 13a | Describe the processes used to decide which studies were eligible for each synthesis (e.g. tabulating the study intervention characteristics and comparing against the planned groups for each synthesis (item #5)). | Page 7 |
|  | 13b | Describe any methods required to prepare the data for presentation or synthesis, such as handling of missing summary statistics, or data conversions. | Page 7 |
|  | 13c | Describe any methods used to tabulate or visually display results of individual studies and syntheses. | Page 7 |
|  | 13d | Describe any methods used to synthesize results and provide a rationale for the choice(s). If meta-analysis was performed, describe the model(s), method(s) to identify the presence and extent of statistical heterogeneity, and software package(s) used. | Page 7 |
|  | 13e | Describe any methods used to explore possible causes of heterogeneity among study results (e.g. subgroup analysis, meta-regression). | Page 8 |
|  | 13f | Describe any sensitivity analyses conducted to assess robustness of the synthesized results. | Page 8 |
| Reporting bias assessment | 14 | Describe any methods used to assess risk of bias due to missing results in a synthesis (arising from reporting biases). | Page 8 |
| Certainty assessment | 15 | Describe any methods used to assess certainty (or confidence) in the body of evidence for an outcome. | Page 8 |
| **RESULTS** | | |  |
| Study selection | 16a | Describe the results of the search and selection process, from the number of records identified in the search to the number of studies included in the review, ideally using a flow diagram. | Page 8 |
|  | 16b | Cite studies that might appear to meet the inclusion criteria, but which were excluded, and explain why they were excluded. | Page 8 |
| Study characteristics | 17 | Cite each included study and present its characteristics. | Page 8 and  Table 1 |
| Risk of bias in studies | 18 | Present assessments of risk of bias for each included study. | Page 12 and Supplementary 3 |
| Results of individual studies | 19 | For all outcomes, present, for each study: (a) summary statistics for each group (where appropriate) and (b) an effect estimate and its precision (e.g. confidence/credible interval), ideally using structured tables or plots. | Page 12 -19 |
| Results of syntheses | 20a | For each synthesis, briefly summarise the characteristics and risk of bias among contributing studies. | Page 13 |
|  | 20b | Present results of all statistical syntheses conducted. If meta-analysis was done, present for each the summary estimate and its precision (e.g. confidence/credible interval) and measures of statistical heterogeneity. If comparing groups, describe the direction of the effect. | Page 13 |
|  | 20c | Present results of all investigations of possible causes of heterogeneity among study results. | Page 12, 16-17 |
|  | 20d | Present results of all sensitivity analyses conducted to assess the robustness of the synthesized results. | Page 16 |
| Reporting biases | 21 | Present assessments of risk of bias due to missing results (arising from reporting biases) for each synthesis assessed. | Page 18 |
| Certainty of evidence | 22 | Present assessments of certainty (or confidence) in the body of evidence for each outcome assessed. | Page 19 |
| **DISCUSSION** | | |  |
| Discussion | 23a | Provide a general interpretation of the results in the context of other evidence. | Page 19 |
|  | 23b | Discuss any limitations of the evidence included in the review. | Page 23 |
|  | 23c | Discuss any limitations of the review processes used. | Page 23 |
|  | 23d | Discuss implications of the results for practice, policy, and future research. | Page 23 |
| **OTHER INFORMATION** | | |  |
| Registration and protocol | 24a | Provide registration information for the review, including register name and registration number, or state that the review was not registered. | Page 5 |
|  | 24b | Indicate where the review protocol can be accessed, or state that a protocol was not prepared. | Page 5 |
|  | 24c | Describe and explain any amendments to information provided at registration or in the protocol. | Page 5 |
| Support | 25 | Describe sources of financial or non-financial support for the review, and the role of the funders or sponsors in the review. | Page 25 |
| Competing interests | 26 | Declare any competing interests of review authors. | Page 25 |
| Availability of data, code and other materials | 27 | Report which of the following are publicly available and where they can be found: template data collection forms; data extracted from included studies; data used for all analyses; analytic code; any other materials used in the review. | Page 25 |

*From:*  Page MJ, McKenzie JE, Bossuyt PM, Boutron I, Hoffmann TC, Mulrow CD, et al. The PRISMA 2020 statement: an updated

guideline for reporting systematic reviews. BMJ 2021;372:n71. doi: 10.1136/bmj.n71. This work is licensed under CC BY 4.0.

To view a copy of this license, visit <https://creativecommons.org/licenses/by/4.0/>

**Supplementary Material 2: Search Strategy**

**Table S1:** Search strategy used for systematic review

| **Search strategy** | **Results** |
| --- | --- |
| **PubMed** |  |
| Search: Retinopathy of Prematurity Filters: from 2010 - 2025 | 6.507 |
| Search: (Retinopathy of Prematurity) AND (anti-VEGF therapy) Filters: from 2010 - 2025 | 354 |
| Search: (Retinopathy of Prematurity) AND (Laser treatment) Filters: from 2010 - 2025 | 1,195 |
| Search: (Retinopathy of Prematurity) AND (Combination therapy) Filters: from 2010 - 2025 | 143 |
| Search: (Retinopathy of Prematurity) AND (ROP regression) Filters: from 2010 - 2025 | 744 |
| Search: (Retinopathy of Prematurity) AND (Visual outcomes) Filters: from 2010 - 2025 | 523 |
| Search: (Retinopathy of Prematurity) AND (bevacizumab) Filters: from 2010 - 2025 | 637 |
| Search: (Retinopathy of Prematurity) AND (ranibizumab) Filters: from 2010 - 2025 | 234 |
| Search: (Retinopathy of Prematurity) AND (aflibercept) Filters: from 2010 - 2025 | 78 |
| **Cochrane Library** |  |
| Retinopathy of Prematurity:ti,ab,kw (Word variations have been searched) | 1,522 |
| Retinopathy of Prematurity:ti,ab,kw AND anti-VEGF therapy:ti,ab,kw (Word variations have been searched) | 22 |
| Retinopathy of Prematurity:ti,ab,kw AND Laser treatment:ti,ab,kw (Word variations have been searched) | 209 |
| Retinopathy of Prematurity:ti,ab,kw AND Combination therapy:ti,ab,kw (Word variations have been searched) | 87 |
| Retinopathy of Prematurity:ti,ab,kw AND regression:ti,ab,kw (Word variations have been searched) | 146 |
| Retinopathy of Prematurity:ti,ab,kw AND Visual outcomes:ti,ab,kw (Word variations have been searched) | 165 |
| Retinopathy of Prematurity:ti,ab,kw AND bevacizumab:ti,ab,kw (Word variations have been searched) | 73 |
| Retinopathy of Prematurity:ti,ab,kw AND ranibizumab:ti,ab,kw (Word variations have been searched) | 44 |
| Retinopathy of Prematurity:ti,ab,kw AND aflibercept:ti,ab,kw (Word variations have been searched) | 16 |
| **Scopus** |  |
| Search document: retinopathy AND of AND prematurity Filters: from 2010 - 2025 | 9,812 |
| Search document: retinopathy AND of AND prematurity AND anti-VEGF AND therapy Filters: from 2010 - 2025 | 335 |
| Search document: retinopathy AND of AND prematurity AND Laser AND therapy Filters: from 2010 - 2025 | 1,398 |
| Search document: retinopathy AND of AND prematurity AND Combination AND therapy Filters: from 2010 - 2025 | 108 |
| Search document: retinopathy AND of AND prematurity AND regression Filters: from 2010 - 2025 | 108 |
| Search document: retinopathy AND of AND prematurity AND visual AND outcome Filters: from 2010 - 2025 | 609 |
| Search document: retinopathy AND of AND prematurity AND bevacizumab Filters: from 2010 - 2025 | 972 |
| Search document: retinopathy AND of AND prematurity AND ranibizumab Filters: from 2010 - 2025 | 434 |
| Search document: retinopathy AND of AND prematurity AND aflibercept Filters: from 2010 - 2025 | 152 |
| **Google Scholar** |  |
| "Retinopathy of Prematurity" AND "anti-VEGF therapy" Custom range 2010-2025 | 3,760 |
| "Retinopathy of Prematurity" AND “Laser treatment for ROP" Custom range 2010-2025 | 7,270 |
| "Retinopathy of Prematurity" AND "Combination therapy" Custom range 2010-2025 | 1,640 |
| "ROP regression" Custom range 2010-2025 | 440 |
| "Visual outcomes of ROP" Custom range 2010-2025 | 12 |
| "Bevacizumab for ROP" Custom range 2010-2025 | 174 |
| "Ranibizumab for ROP " Custom range 2010-2025 | 58 |
| "Aflibercept for ROP " Custom range 2010-2025 | 20 |

**Supplementary Material 3:** Risk of Bias Score for Quantitative Impacts Studies

**Table S2:** Risk of bias assessment of included non-randomized studies using the ROBINS-I tool

| **Study** | **Bias due to confounding** | **Bias in selection of participants** | **Bias in classification of interventions** | **Bias due to deviations from intended interventions** | **Bias due to missing data** | **Bias in measurement of outcomes** | **Bias in selection of reported result** | **Overall risk of bias** |
| --- | --- | --- | --- | --- | --- | --- | --- | --- |
| Isaac 2015 | Moderate | Moderate | Low | High | Low | Moderate | Low | Moderate |
| Morin 2016 | Moderate | Moderate | Low | High | Low | Moderate | Low | Moderate |
| Raghuram 2019 | Moderate | Moderate | Low | High | Low | Moderate | Low | Moderate |
| Hwang 2015 | Moderate | Moderate | Low | High | Low | Moderate | Low | Moderate |
| Gunay 2016 | Moderate | Moderate | Low | High | Low | Moderate | Low | Moderate |
| Kabatas 2017 | Moderate | Moderate | Low | High | Low | Moderate | Low | Moderate |
| Mueller 2017 | Moderate | Moderate | Low | High | Low | Moderate | Low | Moderate |

**Table S3:** Risk of bias assessment of included randomized controlled trials using the RoB 2 tool

| **Study** | **Randomization process** | **Deviations from intended interventions** | **Missing outcome data** | **Measurement of outcome** | **Selection of reported results** | **Overall risk of bias** |
| --- | --- | --- | --- | --- | --- | --- |
| Mintz-Hittner 2011 | Low | Some concerns | Low | Low | Low | Some concerns |
| Zhang 2017 | Low | Some concerns | Low | Low | Low | Some concerns |
| Karkhaneh 2015 | Low | Some concerns | Low | Low | Low | Some concerns |
| Roohipoor 2018 | Low | Some concerns | Low | Low | Low | Some concerns |
| **Lepore 2018** | Low | Some concerns | Low | Low | Low | Some concerns |
| Stahl 2019 | Low | Some concerns | Low | Low | Low | Some concerns |
| Stahl 2022 | Low | Some concerns | Low | Low | Low | Some concerns |
| O’Keeffe 2016 | Low | Some concerns | Low | Low | Low | Some concerns |

**Supplementary Material 4: Certainty of Evidence (GRADE**)

**Table S4:** GRADE (Summary of Findings)

| **Outcome** | **Risk with Laser** | **Risk with Anti-VEGF** | **Relative Effect (95% CI)** | **No. of Participants (Studies)** | **Certainty (GRADE)** | **Comments** |
| --- | --- | --- | --- | --- | --- | --- |
| Recurrence requiring retreatment  (24–90 wks) | 150 per 1000 | 210 per 1000 (120–360) | RR 1.40 (0.80–2.40 | 1,876 eyes (8 studies) | ⊕⊕⊕○  MODERATE | Higher recurrence trend with anti-VEGF; moderate certainty. |
| Time to recurrence  (3–13 wks) | Mean 10.5 wks | 2.8 wks shorter (4.2 to 1.4 shorter) | MD −2.8 wks   (−4.2 to −1.4 | 344 eyes (4 studies) | ⊕⊕○○  LOW | Earlier recurrence after anti-VEGF; substantial heterogeneity. |
| Retreatment rate  (24–90 wks) | 88 per 1000 | 127 per 1000 (70–230) | RR 1.44 (0.80–2.60 | 1,986 eyes (6 studies) | ⊕⊕○○  LOW | Uncertain effect; wide CI and study limitations. |
| High myopia (≥−5 D) (2–5 yrs) | 200 per 1000 | 80 per 1000 (40–160) | RR 0.40 (0.20–0.80 | 394 eyes (3 studies) | ⊕⊕⊕○ MODERATE | Anti-VEGF markedly lowers high myopia risk. |
| Unfavorable structural outcome  (24 wks–5 yrs) | 134 per 1000 | 67 per 1000 (40–114) | RR 0.50 (0.30–0.85 | 1,456 eyes (6 studies) | ⊕⊕⊕○ MODERATE | Better structural outcomes with anti-VEGF. |
